# Ampullary Carcinoma: An Overview of a Rare Entity and Discussion of Current and Future Therapeutic Challenges

**DOI:** 10.3390/curroncol28050293

**Published:** 2021-09-01

**Authors:** Alessandro Rizzo, Vincenzo Dadduzio, Lucia Lombardi, Angela Dalia Ricci, Gennaro Gadaleta-Caldarola

**Affiliations:** 1 Medical Oncology, IRCCS Azienda Ospedaliero-Universitaria di Bologna, Via Albertoni-15, 40128 Bologna, Italy; Dalia.ricci@aslbat.it; 2 Medical Oncology Unit, “Mons. R. Dimiccoli” Hospital, 76121 Barletta (BT), ASL BT, Italy; Vincenzo.dadduzio@aslbat.it (V.D.); Lucia.Lombardi@aslbat.it (L.L.); Gennaro.gadaleta@aslbat.it (G.G.-C.)

**Keywords:** ampullary cancer, ampulla of Vater cancer, biliary tract cancer, chemotherapy, adenocarcinoma

## Abstract

Ampullary carcinomas (ACs) represent a rare entity, accounting for approximately 0.2% of all gastrointestinal solid tumors and 20% of all periampullary cancers (PACs). Unfortunately, few data are available regarding the optimal therapeutic strategy for ACs due to their rarity, and physicians frequently encounter significant difficulties in the management of these malignancies. In this review, we will provide an overview of current evidence on AC, especially focusing on biological features, histological characteristics, and available data guiding present and future therapeutic strategies for these rare, and still barely known, tumors.

## 1. Introduction

**Figure 1:**
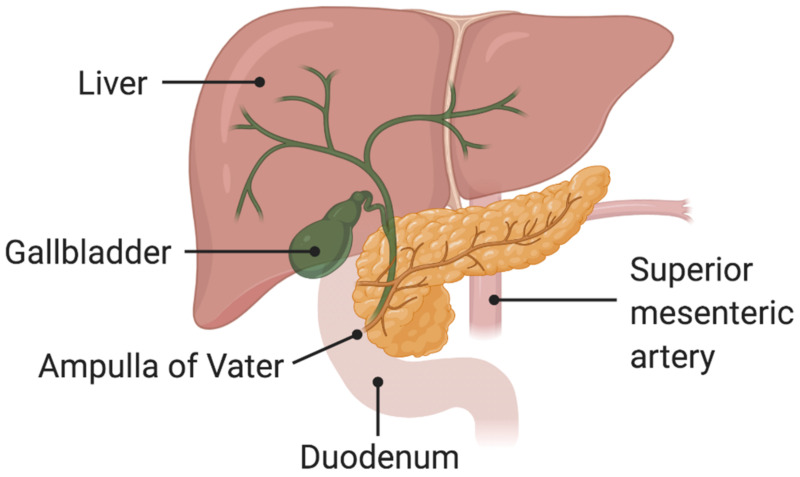
Schematic representation of the ampulla of Vater.

Ampullary carcinomas (ACs), or ampulla of Vater carcinomas, represent 0.2% of all gastrointestinal tumors and account for approximately 20% of all periampullary cancers (PACs) [1,2]. ACs have been suggested to be slightly more frequent in male patients, with a wide age range at diagnosis [1,3]. Although the overall incidence of ACs in Western countries is less than 0.5 cases per 100,000 individuals according to data from international registries, the incidence rate of ACs has shown a significant increase over the last decades, due to the growing use of upper endoscopy for other unrelated indications and the screening of high-risk patients with familiar adenomatous polyposis (FAP) [3,4]. From an anatomical point of view, AC develops from the ampulla of Vater, the “trait d’union” between the main pancreatic duct and the distal common bile duct (CBD), representing a landmark between the hindgut and the foregut (Figure 1) [4,5]. The eponymic term of the ampulla derives from the German anatomist Abraham Vater who first described this mucosal papillary mound in 1720 [5,6]. On the basis of its localization, the majority of patients with AC present with jaundice and other symptoms and signs similar to those of distal cholangiocarcinoma and pancreatic head adenocarcinoma [5,6].

In this paper, we provide a comprehensive overview of available evidence regarding ACs, especially focusing on key aspects involved in the management of these rare malignancies. We performed research on Pubmed/Medline, Cochrane library and Scopus using the keywords “ampullary cancer” OR “ampulla of Vater” OR “ampullary carcinoma” OR “biliary tract cancer”. We selected the most relevant and pertinent studies considering quality of the studies in terms of their applicability, how they were conducted, statistical analysis, number of patients enrolled, and outcomes. A total of 178 potentially relevant reports were identified, which were restricted to 67 following independent evaluation of three authors. We excluded 111 records as non-pertinent reports.

## 2. Histological Features

The majority of ACs consists of adenocarcinomas, with two main different histologic subtypes described according to their epithelium of origin: intestinal- and pancreaticobiliary-type ACs [6,7]. In 1994, a landmark study conducted by Kimura and colleagues was the first to highlight the presence of these two subtypes, with the intestinal form that was suggested to originate from the intestinal epithelium above the ampulla, and pancreaticobiliary-type AC from the epithelium of the distal pancreatic duct and the CBD [8]. Following the results of this study, ACs have been classified into these two forms. More recently, in 2010, the World Health Organization (WHO) revised this classification and the criteria for the histological diagnosis of ACs, introducing a third subtype [9]. In fact, the current classification includes intestinal-type ACs, pancreaticobiliary-type ACs, and mixed-type ACs [9,10]. Of note, intestinal-type ACs have been historically associated with a less aggressive clinical course compared to pancreaticobiliary-type malignancies, with a median overall survival of approximately 16 and 115 months, respectively (5-year survival rate 5–36% versus 50–100%) [10,11]; however, this suggestion has not been confirmed in recent studies, which failed to find prognostic differences between the two groups [12,13].

From a histological point of view, intestinal-type AC is usually characterized by the presence of a non-invasive component of duodenal adenoma, and the morphology of this subtype has been suggested to be similar to that of colorectal cancer, with central necrosis and cribriform or tubular glands [13,14]. Classically, intestinal-type ACs present a smaller invasive component and less frequent perineural and lymphovascular invasion, something that has been related to a better prognosis compared to pancreaticobiliary-type ACs [15,16]. In terms of immunohistochemistry, intestinal-type AC frequently presents the expression of cytokeratin 20 (CK20), mucin 2 (MUC2), caudal-related homeodomain transcription factor 2 (CDX2) and other classically intestinal markers [15,16]; conversely, the pancreaticobiliary subtype has important immunohistochemical analogies with distal cholangiocarcinoma and pancreatic adenocarcinoma, due to the presence of abundant desmoplastic stroma and atypical cells [15,16,17], and the frequent immunohistochemical expression of mucin 1 (MUC1), cytokeratin 7 (CK7), and mucin 5AC (MUC5AC) [15,16,17].

However, a proportion of ACs ranging from 20 to 40% can show features not definitely attributable to a single phenotype [16,17,18]. The overlapping between pancreaticobiliary and intestinal features is relatively common in ACs, leading to the identification of a third “entity”: the mixed-type AC. Further complicating the scenario, there is currently no clear and unambiguous definition of mixed-type AC, with different authors proposing distinct definitions for the mixed subtype [18,19]; for example, Chang and colleagues proposed the definition of the mixed-type subtype for all those ACs presenting at least or more than 10% of both histologic subtypes [20]. Conversely, other authors including Ang supported the presence of at least 25% of both histology types to define AC as “mixed” or, alternatively, an AC comprised entirely of hybrid features [21]. Several pathologists have supported the role of hematoxylin and eosin (HE) staining in guiding the diagnosis of mixed-type AC, as witnessed by the results observed by using a four-marker panel including MUC1, MUC2, CK20, and CDX2 [22,23].

In terms of prognosis, the previously cited study conducted by Chang et al. suggested for mixed-type ACs an intermediate prognosis between the intestinal and the pancreaticobiliary subtypes [20]. Conversely, Asano and colleagues evidenced a similar prognosis in mixed-type and pancreaticobiliary ACs, suggesting that clinical outcomes in patients with the mixed subtype could be more similar to those with pancreaticobiliary-type AC [24].

## 3. Genetic and Molecular Features

Several studies suggested that ACs could present the adenoma–carcinoma sequence, as observed in other gastrointestinal malignancies such as colorectal cancer [25,26]. Although most ACs occur sporadically and in absence of a clearly identifiable etiology, the presence of FAP has been associated with a 200-fold higher risk of AC compared with the general population [26,27].

In terms of molecular features, several recent studies have provided an impressive amount of molecular information regarding hepatobiliopancreatic tumors, including ACs [28,29,30,31,32,33]. Two landmark studies conducted by Yachida and Gingras highlighted the presence of specific mutations, such as *APC*, *SMAD4*, *TP53*, and *KRAS* (Table 1) [34,35]. Moreover, these two reports have studied putative associations between gene mutations and histological subtypes, with an increased incidence of *KRAS*, *TP53*, and *SMAD4* mutations and *APC*, *TP53*, and *KRAS* mutations in the pancreaticobiliary-type and the intestinal-type ACs, respectively (Table 1) [34,35].

Conversely, the most common mutations found in the mixed subtype were *KRAS*, *APC*, and *TP53* (49%, 50%, and 41%, respectively, according to the results observed by Gingras and colleagues) [34,35]. These studies have also suggested an important role in the carcinogenesis process of AC played by inactivating mutations of *ELF3*, since these aberrations have been observed in approximately the 11% of cases of AC [34,35,36]. According to the results of the previously cited paper by Yachida and colleagues, the tumor suppressor gene *ELF3* seems to act as an AC driver due to its role in the regulation of epithelial differentiation. However, these findings are still preliminary and further studies are warranted.

In the last few years, clinicians and researchers have been “tempted” to base their therapeutic decisions on the basis of molecular profiling, since some features have been suggested to have a putative prognostic value. In particular, a study by Mafficini et al. linked the presence of *KRAS* and *TP53* mutations with a more aggressive clinical course, regardless of histology, with these mutations representing drivers of early cancer progression [37]. Other aberrations, including the Wnt-pathway and erythroblastosis oncogene B (*ERBB*) mutations, have been suggested to occur at later stages, with the latter having the potential to represent a therapeutic target for molecularly driven therapies [38,39,40]. Similarly, the assessment of microsatellite instability (MSI) could be useful to guide the treatment algorithm towards the use of immune checkpoint inhibitors, given the already proven high susceptibility to immunotherapy of MSI high patients, regardless of histology and primary tumor site [41,42,43,44]. However, few data are available on the prevalence of MSI in AC patients [43,44].

## 4. Clinical Features, Differential Diagnosis and Staging

ACs usually present symptoms and signs frequently observed in extrahepatic cholangiocarcinoma and pancreatic adenocarcinoma [45,46]: jaundice, diarrhea, steatorrhea, and gastrointestinal bleeding with melena are frequent findings and the malignancy is commonly diagnosed early due to these symptoms, occurring earlier than, for example, in pancreatic cancers [45,46]. Endoscopic ultrasound (EUS), endoscopic retrograde cholangiopancreatography (ERCP) and fine-needle aspiration cytology (FNAC) are the modalities of choice for diagnosis, while computed tomography (CT) of chest, abdomen, and pelvis are fundamental tools to stage AC [46,47]. Endoscopic ampullectomy is considered as an integral part of the diagnostic process and may also be curative in highly selected cases—including early AC, in situ carcinoma or low-grade dysplasia. In terms of differential diagnosis, primary ACs must be distinguished from pancreatic cancers, distal cholangiocarcinoma, and small bowel cancers [48,49]. At the same time, FNAC frequently encounters difficulties in differentiating ACs from other PACs, and thus, only definitive surgery may lead to appropriate diagnosis [48,49].

The peculiar anatomical location of the ampulla makes tumor staging particularly complex [48,49]. According to the 8th edition of the TNM staging system by the Union for International Cancer Control (UICC), the T stage is subclassified on the basis of the depth of duodenum and pancreas invasion [49]. In particular, T1 is split into T1a and T1b, if the malignancy is limited to the sphincter of Oddi or if it invades the duodenal submucosa, respectively (Table 2) [49]; similarly, T3a identifies an AC reporting an invasion into the pancreas equal or less than 0.5 cm, while T3b ACs invade the pancreas for more than 0.5 cm or report an invasion of the duodenal subserosa. The involvement of the celiac axis, the superior mesenteric artery, and/or the common hepatic artery defines the T4 stage [49]. While the 7th edition only identified the presence/absence of nodal involvement, the 8th edition of the UICC classification and staging system has split the N classification into N1, in the case of 1–3 positive lymph nodes, and N2 (4 or more) (Table 2) [49]. Some authors recently raised some concerns about this novel classification: Kim and colleagues suggested that the T category is not able to properly classify and stratify AC patients according to their prognosis [50]; similarly, Imamura and colleagues critically discussed the effective prognostic accuracy of this staging classification, suggesting that the 8th edition UICC classification may be associated with poor risk stratification, showing that the novel subcategories did not improve the prognostic accuracy [51].

## 5. Surgery

Radical surgical resection with lymphadenectomy represents the mainstay of treatment for AC, and pancreaticoduodenectomy (PD) is the current standard, with curative surgery that is deemed possible in approximately 50% of cases [52,53]. Given the high rate of lymph nodes involved (around 30–40%), especially those located around the superior mesenteric artery and pancreatico-duodenal sites, adequate lymphadenectomy with the dissection of at least 12 lymph nodes is of pivotal importance [53,54]. In addition, despite the high resectability rate observed for AC patients receiving laparotomic surgery, the postoperative course is frequently burdened by a higher proportion of complications compared to pancreatic adenocarcinoma [53,54]. Thus, several minimally invasive modalities have been explored, including robot-assisted and laparoscopic surgery. However, the real benefit of these approaches remains unclear [55,56]; a recent systematic review and meta-analysis published by Chen and colleagues compared minimally invasive PD versus conventional open PD across 3402 patients with ACs or PACs [57]. According to the findings of this report, the authors observed that minimally invasive surgery was associated with statistically significant lower transfusion rate and blood loss as well as shorter hospital stay [57]. Conversely, no differences were highlighted in terms of readmission, number of retrieved lymph nodes and post-operative complications [57]. However, minimally invasive PD is still limited to tertiary referral centers with adequate expertise, and prospective clinical trials are required to corroborate these results [55,56].

Alternative endoscopic approaches have been also studied, and in selected early-stage cases endoscopic ampullectomy is preferred as an alternative to conventional open PD, especially for selected patients with noninvasive forms, also considering the lower procedure-related morbidity [58,59]. However, due to the frequent local and locoregional lymph nodes involvement, this technique is rarely used [60,61]. According to previous studies, higher risk of relapse and worse clinical outcomes have been associated with pancreatic invasion, lymph nodes involvement, and poor differentiation (G3) [61,62]. Other negative prognostic factors are represented by T3b and T4 disease, tumor size and tumor involvement of resection margins [61,62]. In regard to the latter, tumor involvement of resection margins has been suggested to play an important role as an adverse prognostic factor in comparison with negative margin resections, being associated with a median survival of approximately 60 months versus 12 months, respectively, as reported in a landmark study conducted by Howe and colleagues [61,62].

## 6. Medical Treatment

Although radical surgery is a curative modality for early-stage AC, approximately half of patients develop tumor recurrence [61,62,63]. Thus, postoperative approaches including chemotherapy, radiotherapy or chemoradiation have been evaluated over the years [62,63]. Nonetheless, data regarding adjuvant systemic chemotherapy are limited to retrospective studies, with no randomized clinical trial conducted in AC patients so far due to several reasons, including the rarity of the disease and frequent misclassifications. In a propensity score-matched analysis, Nassour and colleagues highlighted that adjuvant chemotherapy was associated with improved survival in 4190 AC patients from the National Cancer Database [64]; in particular, a median overall survival of 47.2 months was reported in the chemotherapy arm compared to 35.5 months in the observation alone group [64]. Similarly, adjuvant chemoradiation therapy reported improved overall survival compared to observation, and this benefit was more important in patients with higher T and/or N [64].

Notably enough, adjuvant and first-line treatment are frequently tailored according to the histologic subtype, and thus, pancreaticobiliary-like ACs are commonly treated like pancreatic adenocarcinoma or biliary tract cancer [65]. Conversely, patients with intestinal-like ACs receive regimens typically used for colorectal cancer. However, a retrospective study conducted by Ecker and colleagues found that the use of adjuvant chemotherapy was not associated to improved clinical outcomes either for intestinal nor for pancreaticobiliary ACs [65]. Based on these premises and given the lack of level 1 evidence supporting the use of adjuvant chemotherapy and chemoradiotherapy, the optimal regimen for AC both in adjuvant and metastatic settings remains to be defined. In a study conducted by Bolm and colleagues including 214 patients, pancreatobiliary-like ACs receiving adjuvant therapy reported an improved median overall survival (adjuvant therapy 85 months versus no adjuvant treatment 65 months); moreover, adjuvant therapy was suggested to represent an independent prognostic factor in multivariate analysis. Conversely, no benefit was reported in intestinal subtype AC patients, suggesting that adjuvant treatment could be indicated in pancreatobiliary- or mixed-type ACs [66]. In another recent study by Affi Koprowski et al. including 53 patients, stage II and III disease was associated with lower survival, and the use of adjuvant chemotherapy reported improved outcomes in this patient population. Based on these results, the authors suggested that stage could represent a primary determinant of clinical outcomes in AC patients, which could be improved by the use of adjuvant treatment [67]. In addition, some results may be extracted from landmark clinical trials evaluating systemic chemotherapy in biliary tract cancer trials including ACs [68,69]. In the case of advanced AC with distant metastases, systemic chemotherapy represents the gold standard. A historical, long-standing issue in these studies was the inclusion of several different molecular and anatomical subgroups, such as intrahepatic cholangiocarcinoma, extrahepatic cholangiocarcinoma, gallbladder cancer, and even AC [69,70]. For example, the landmark ABC-02 trial which established gemcitabine plus cisplatin as the reference doublet for first-line treatment in biliary tract cancer, included 11 and 9 AC patients in the experimental arm (gemcitabine-cisplatin) and the control arm (gemcitabine alone), respectively [71,72]. Similarly, the recently published ABC-06 trial conducted by Lamarca and colleagues supporting the use of mFOLFOX plus active symptom control (ASC) as second-line treatment in biliary tract cancer, included 11 cases of AC in the experimental arm [73]. In particular, looking at the survival rates for different histologic subgroups, the benefit provided by mFOLFOX suggests a particular activity in AC patients, as witnessed by a median overall survival of 10.4 months (95% Confidence Interval [CI], 9.8—not reached) in ACs compared with 5.7 months (95% CI, 4.1–7.4) in intrahepatic cholangiocarcinoma patients [73,74]. However, the small sample of this population cannot lead to draw any conclusion on efficacy [73,74].

Recent years have seen the advent of next-generation sequencing (NGS) in solid malignancies, leading to the identification of previously unknown molecular data and the emerging of an impressive number of specific aberrations, such as fibroblast growth factor receptor 2 (*FGFR-2*) gene fusions or rearrangements and isocitrate dehydrogenease-1 (*IDH-1*) mutations in intrahepatic cholangiocarcinoma [75,76,77,78,79,80,81,82,83]. In fact, evolution in sequencing technology has enabled the use of genetic information to direct targeted therapies, also leading to a better understanding of cholangiocarcinoma carcinogenesis [75,76,77,78,79,80,81,82,83]. Thus, current and future studies are focusing their attention on the molecular features of ACs, trying to translate the results observed in other biliopancreatic malignancies [37,38]. For example, since *WNT* pathways and *PI3K* alterations have been reported in more than 30% of all ACs, regardless of the histological subtype, and since everolimus, temsirolimus and other agents have shown to be effective in the case of PI3K mutations, these anticancer drugs are under evaluation also in ACs [37,38]. Large-scale, prospective randomized clinical trials are eagerly awaited to further clarify the role of systemic treatment in this setting.

## 7. Conclusions

ACs are rare malignancies arising from the ampullary complex, distal to the confluence of the pancreatic duct and the common bile duct [1,2]. As previously stated, and in contrast to other malignancies, ACs usually present earlier in their disease course [2,3]. The management of ACs remains hampered by several elements, including the paucity of data regarding the correlation between prognosis and systemic treatment, the lack of a comprehensive molecular classification, and the presence of issues regarding current histological classification [6,7]. Despite recent years having witnessed the advent of novel therapeutic options in several tumor types, efforts in rare malignancies including ACs remains challenging since few evidence-based data are available so far. At this time, available literature is mainly represented by retrospective studies and subgroup analysis of randomized, multicenter trials. In our opinion, it remains unlikely to perform randomized controlled clinical trials adequately powered to comprehend the role of several treatment modalities in this setting. Further efforts are needed in order to better stratify AC patients and to improve the natural history of this rare, and still barely known, group of gastrointestinal malignancies with many unanswered questions.

## Figures and Tables

**Table 1:** The more frequently observed gene mutations in ampullary cancer, according to recent studies conducted by Yachida and Gingras [34,35].

Author (Reference)	Intestinal-Type Ampullary Cancer (Percentage)	Pancreaticobiliary-Type Ampullary Cancer (Percentage)	Mixed-Type Ampullary Cancer (Percentage)
Yachida [34]	*APC* (49%)	*KRAS* (67%)	
	*TP53* (39%)	*TP53* (67%)	
	*KRAS* (39%)	*SMAD4* (20%)	
	*CTNNB1* (26%)	*CTNNB1* (15%)	
	*ARID2* (18%)	*ERBB3* (14%)	
Gingras [35]	*TP53* (64%)	*TP53* (71%)	*KRAS* (49%)
	*KRAS* (46%)	*KRAS* (65%)	*APC* (50%)
	*APC* (41%)	*SMAD4* (18%)	*TP53* (41%)
	*PIK3CA* (26%)	*CDKN2A* (16%)	*SMARCA4* (27%)
	*SMAD4* (20%)	*PIK3CA* (13%)	*PIK3CA* (23%)

**Table 2:** The 8th edition of the TNM staging system by the Union for International Cancer Control (UICC).

**T**	TX: primary tumor cannot be assessed
	T0: no evidence of primary tumor
	T1a: limited to sphincter of Oddi
	T1b: invasion into duodenal submucosa
	T2: invasion into duodenal muscularis propria
	T3a: invasion into pancreas ≤ 0.5 cm
	T3b: invasion into pancreas > 0.5 cm
	T4: involvement of celiac or superior mesenteric artery
**N**	NX: lymph nodes cannot be assessed
	N0: no lymph node involvement
	N1: metastasis in 1–3 lymph nodes
	N2: metastasis in 4 or more lymph nodes
**M**	MX: distant metastasis cannot be assessed
	M0: no distant metastasis
	M1: distant metastasis
